# Unique genetic loci identified for emotional behavior in control and chronic stress conditions

**DOI:** 10.3389/fnbeh.2014.00341

**Published:** 2014-10-21

**Authors:** Kimberly A. K. Carhuatanta, Chloe J. A. Shea, James P. Herman, Ryan Jankord

**Affiliations:** ^1^Applied Neuroscience, 711th Human Performance Wing, Air Force Research LaboratoryWright-Patterson Air Force Base, OH, USA; ^2^Research Associate Program, National Research Council, National Academies of SciencesWashington, DC, USA; ^3^Department of Psychiatry and Behavioral Neuroscience, University of CincinnatiCincinnati, OH, USA

**Keywords:** BXD, QTL, stress, fear learning, anxiety, genetics, emotional behavior

## Abstract

An individual's genetic background affects their emotional behavior and response to stress. Although studies have been conducted to identify genetic predictors for emotional behavior or stress response, it remains unknown how prior stress history alters the interaction between an individual's genome and their emotional behavior. Therefore, the purpose of this study is to identify chromosomal regions that affect emotional behavior and are sensitive to stress exposure. We utilized the BXD behavioral genetics mouse model to identify chromosomal regions that predict fear learning and emotional behavior following exposure to a control or chronic stress environment. 62 BXD recombinant inbred strains and C57BL/6 and DBA/2 parental strains underwent behavioral testing including a classical fear conditioning paradigm and the elevated plus maze. Distinct quantitative trait loci (QTLs) were identified for emotional learning, anxiety and locomotion in control and chronic stress populations. Candidate genes, including those with already known functions in learning and stress were found to reside within the identified QTLs. Our data suggest that chronic stress history reveals novel genetic predictors of emotional behavior.

## Introduction

Much of an individual's emotional behavior and response to stress is determined by genetic factors. Mood and anxiety disorders are highly heritable (Bienvenu et al., 2011; Binder, 2012). Environmental stressors often exacerbate psychiatric disease in individuals diagnosed with mood and anxiety disorders, resulting in attention deficits, difficulty in memory tasks, irritability, and affect changes in humans (Renoir et al., 2013). In rodents, chronic stress, and hypothalamus-pituitary- adrenal axis (HPA) activation result in anhedonia, sleep abnormalities, circadian disturbances, and memory impairment (Herman and Watson, 1995; Pardon et al., 2000a,b; de Kloet et al., 2005). Additionally, emotional learning, and anxiety/exploratory behavior are subject to stress-induced effects (Mozhui et al., 2010).

While the effects of both genetics and environment on emotional behavior have been described (Valentinuzzi et al., 1998; Mozhui et al., 2010), to date, the interplay between all three (i.e., genetics, environment, and emotional behavior) has been less studied. In humans, the interaction of genetic factors and chronic stress has been investigated in populations having high stress lifestyles or occupations such as medical or nursing residents and military or fire-fighting service positions or low socioeconomic status (Reijneveld and Schene, 1998; Chernomas and Shapiro, 2013; Powell et al., 2013). For in depth genetic analysis of behavior, researchers can rely on recombinant inbred strains, such as the BXD mouse strain populations (Philip et al., 2010). The BXD mouse population is a behavioral genetics model of recombinant inbred mouse strains derived from the C57BL/6 and DBA/2 parental strains, and is used by researchers to map phenotypic variation across RI BXD strains onto defined chromosomal regions via quantitative trait loci (QTL) analyses (Peirce et al., 2004; Andreux et al., 2012). Based on the differences in performance and stress responsiveness between C57BL/6 and DBA/2 strains (Waddell et al., 2004; Brigman et al., 2009; Mozhui et al., 2010), the BXD mouse population provides a unique opportunity to identify the chromosomal locations that contribute to emotional behavior.

Here, we test the hypothesis that environmental context (the presence or absence of stress) reveals unique genetic predictors of emotional behavior. Emotional behavior of 62 BXD strains, and C57BL/6 and DBA/2 parental strains was assessed by fear conditioning and performance on the elevated plus maze. Littermates of each strain were treated with either control or chronic variable stress (CVS). QTL mapping was performed to identify genetic loci and candidate genes underlying emotional learning and behavior in both the control and chronic stress states.

## Materials and methods

### Ethics statement

All animal procedures were approved by the Air Force Research Laboratory Institutional Animal Care and Use Committee in accordance with the NIH Guide for the Care and Use of Laboratory Animals.

### Animals

All BXD recombinant inbred strains and their parental C57BJ/6 and DBA/2 strains were purchased in littermate pairs from Jackson Laboratory (Bar Harbor, ME, USA). Male mice, aged 9 weeks at start of experiments, were singly housed with *ad libitum* access to standard rodent chow (Harlan Teklad, Madison, WI, USA) and water. Ambient housing conditions were controlled for temperature (18–24°C, 21°C average), humidity (30–70%, 35% average), and the housing room maintained a standard 12:12 light cycle (6 a.m. on). Sixty-two different BXD strains (592 BXD mice, *n* = 3–5 per strain per group), in addition to the C57BJ/6 and DBA/2 parental strains (*n* = 5 per strain per group), were utilized in the study.

### Experimental design

**Figure 1:**
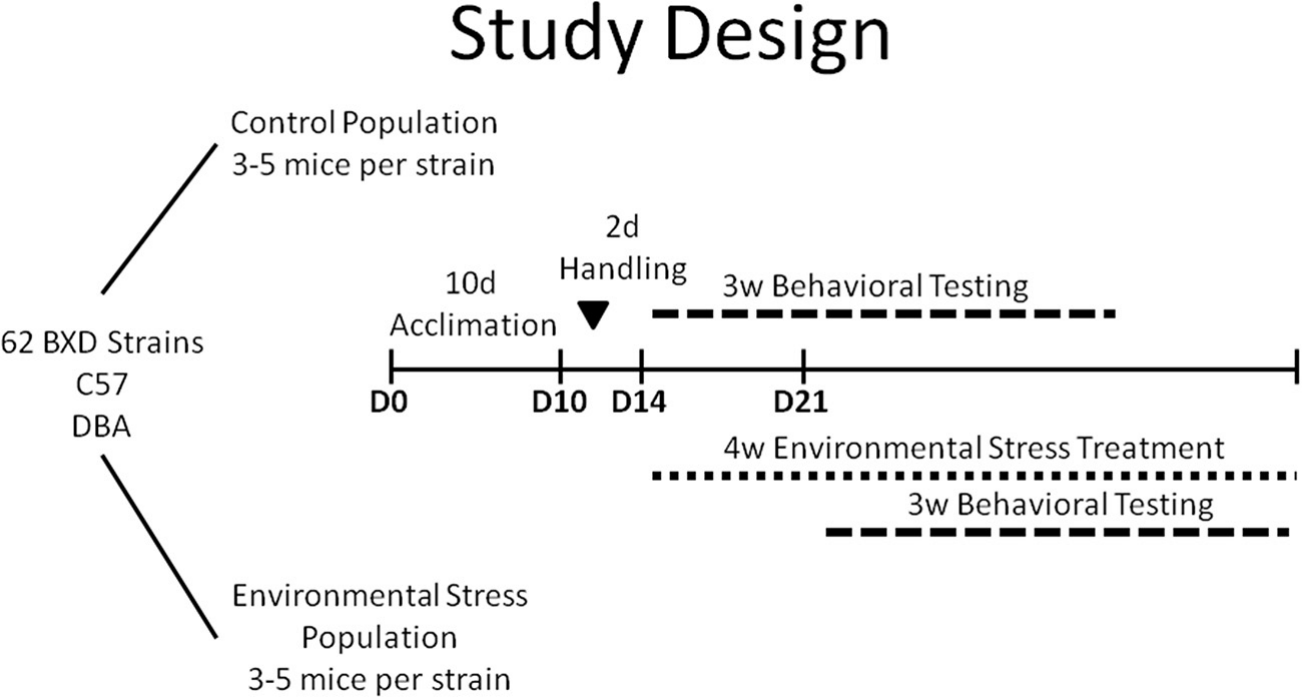
**Study Design**.

The design of this study is summarized in Figure 1. Upon arrival at the animal facility, littermate pairs were divided into control and chronic stress (CVS) populations and were allowed to acclimate to our animal facility and to single housing for a 10 days period. Both populations of animals were housed in the same room. All treatment and testing occurred during Monday–Friday of the week during the light cycle. After 2 days of handling, the control population began behavioral testing (weeks 1–3) and the CVS population began the chronic stress paradigm (weeks 1–4). The CVS paradigm lasted 4 weeks with behavioral testing being completed during weeks 2–4 for CVS mice. Behavioral testing in the chronic stress population was offset for 1 week from environmental controls to allow 1 week of exposure to the CVS paradigm before behavioral testing and to accommodate throughput limitations of the behavioral assays. All mice underwent 3 weeks of behavioral testing such that the elevated plus maze (EPM) task preceded the learning tasks. Following the EPM, mice complete a 7 days Morris water maze (data not described in this manuscript), followed by the fear conditioning test. The order of tests permitted the observation of anxiety-like behavior prior to any conditioning effects. Following the completion of all behavioral tests, animals were euthanized via rapid decapitation.

Ten cohorts (50–64 mice) of BXD strains (25–32 strains per cohort) were utilized over the span of a year. Each cohort consisted of two littermates of each strain and had a unique grouping of BXD strains to minimize confounding factors such as seasonal and group effects.

### Chronic stress treatment

Chronic stress was established using the CVS model (Herman and Watson, 1995; Furay et al., 2008; Castañeda et al., 2011). In this paradigm, animals underwent a randomized schedule of one to two mild to moderate physiological and psychological stressors daily 5 days a week. Stressors included novel overnight home cage, hypoxia (30 min), cold exposure (15 min at 4°C), open field (30 min), and constant motion (60 min) exposure. The randomization of the schedule resulted in unique order and sequence of the stressors thereby limiting predictability and habituation to the chronic stress paradigm. All cohorts of animals were exposed to the same order and sequence of stressors.

### Elevated plus maze (EPM)

Prior to any additional behavioral testing, mice were subjected to the EPM in which they were placed in the center of the EPM apparatus, and recorded and monitored using EthoVision XT software (Noldus Information Technologies, Leesburg, VT) for 5 min. Experimenter did not remain in EPM testing area during data collection. The EPM is elevated 1 m above the floor and consists of two open and closed arms (40 × 8 cm) separated by a center area. Closed arms contain vertical gray plexiglass walls (29.5 cm), while open arms contain no protective edges. During testing, the maze is illuminated by two 60 W incandescent light bulbs placed approximately 35 cm above the arms in a room illuminated by two shielded 150 W incandescent light bulbs. The measures collected via EthoVisionXT software (Nolud Information Technologies, Leesburg, VT) from the EPM test include: (1) time spent in the closed arms, (2) time spent in the open arm, and (3) distance traversed throughout the maze. Time spent in the closed arms has been associated with anxiety-like behavior, while time in open arms is considered exploratory. Distance traversed was assessed as a measure of locomotor activity within BXD strains.

### Fear conditioning paradigm

Experiments were completed using four fear conditioning chambers (Med Associates, Inc., St. Albans, VT, USA). Chambers consist of front and back Plexiglas and side metal walls. Fear conditioning chambers were housed within camera containing sound attenuation cubicles that reduce outside sound and light during testing. Fear Conditioning was completed over 3 days. On day 1, mice were placed into the operant chambers (with steel grid floor) and freezing behavior is assessed using EthoVision XT software (Noldus Information Technologies, Leesburg, VT) throughout the session. Day 1 session included four exposures to a 30 s tone (85 dB, 3 KHz) that terminated with a 2 s foot-shock (0.75 mA), which elicited freezing behavior (unconditioned response) that was captured via EthoVision software. Each tone was separated by a 30 s inter-tone interval. The pairing of the tone and shock (unconditioned stimulus, US) on Day 1 resulted in the establishment of the tone as a conditioned stimulus (CS), and elicited freezing behavior (conditioned response). On Day 2, mice were returned to the operant chamber and freezing behavior was monitored for 10 min. On Day 3, the environmental context of the operant chamber was altered by replacing the grid floor with a smooth white Plexiglas insert. Mice were then exposed to the 30 s CS (tone) 9 times with random inter-tone intervals.

We measured the percent time displaying freezing behavior on Day 1 during acquisition (180 s following the final exposure to the CS-US pairing), Day 2 after placement within the conditioning context (180 s), and Day 3 during the presentation of the auditory cue (30 s).

#### Statistics

Stress-effect was calculated as the difference in performance between control and CVS littermates. Mixed model analysis was performed via lme4 and lmerTest packages in R using stress, strain, month of testing, and experimenter handling during testing as fixed variables and cohort as a random variable. Non-linear mixed-effect test was followed by ANOVA to obtain values for main effects of fixed variables. Pearson product-moment correlations (R) and Spearman rank order correlations (*rho*) between traits measured were computed via GeneNetwork for both the Control and Stress population.

#### Summary statistics of heritability of behavioral traits measured in FC and EPM testing

Broad-sense (*H*^2^) and narrow-sense (*h*^2^) heritability of expression levels in the recombinant inbred lines was estimated using the Hegmann and Possidente Method (1981): *H*^2^ = Va/Vt; *h*^2^ = ½Va/(½Va + Vw). Va is the variance among strains, Vt is the variance in the total population, and Vw is the variance within strains.

### QTL mapping

QTL mapping was performed using complex trait analysis and mapping tools available on the Genenetwork website (http://www.genenetwork.org). GeneNetwork utilizes 3806 markers (intermarker interval of 0.66 Mb) in 89 BXD recombinant inbred strains to link regions of the genome to differences in phenotype. Potential QTLs are evaluated at regular intervals along the genome, each evaluated for significance via 2000 permutation tests (Churchill and Doerge, 1994). Thresholds for suggestive and significant QTLs are determined via GeneNetwork as likelihood ratio statistic (LRS) values associated with genome-wide probabilities of 0.67 and 0.05, respectively (Williams et al., 2001). QTLs are described here by their greatest LRS value, significance threshold passed, confidence interval (evaluated using the 1 LOD drop method; Lander and Botstein, 1989), and by the number of genes residing within the described peak. Mapping was performed for three traits of emotional learning assessed during the fear conditioning paradigm (freezing during training to tone and shock on Day 1, freezing to environmental context on Day 2, and freezing to tone on Day 3) and three emotional behavioral traits measured within the elevated plus maze [locomotion or distance (m) traversed, time (s) in closed arms, and time (s) in open arms].

### Candidate genes

Candidate genes listed are genes within each QTL which have a human homolog and/or are cis-regulating. Determination of cis-regulation was performed by using the QTLminer tool of GeneNetwork to identify cis-regulated genes (amygdala, cerebellum, hippocampus, hypothalamus, neocortex, prefrontal cortex, or striatum) within the confidence interval of each QTL, Genes were further assessed via literary search in PubMed for known relationship with stress, learning, anxiety, fear conditioning, and exploration.

## Results

### Emotional behavior in BXD mice

**Figure 2:**
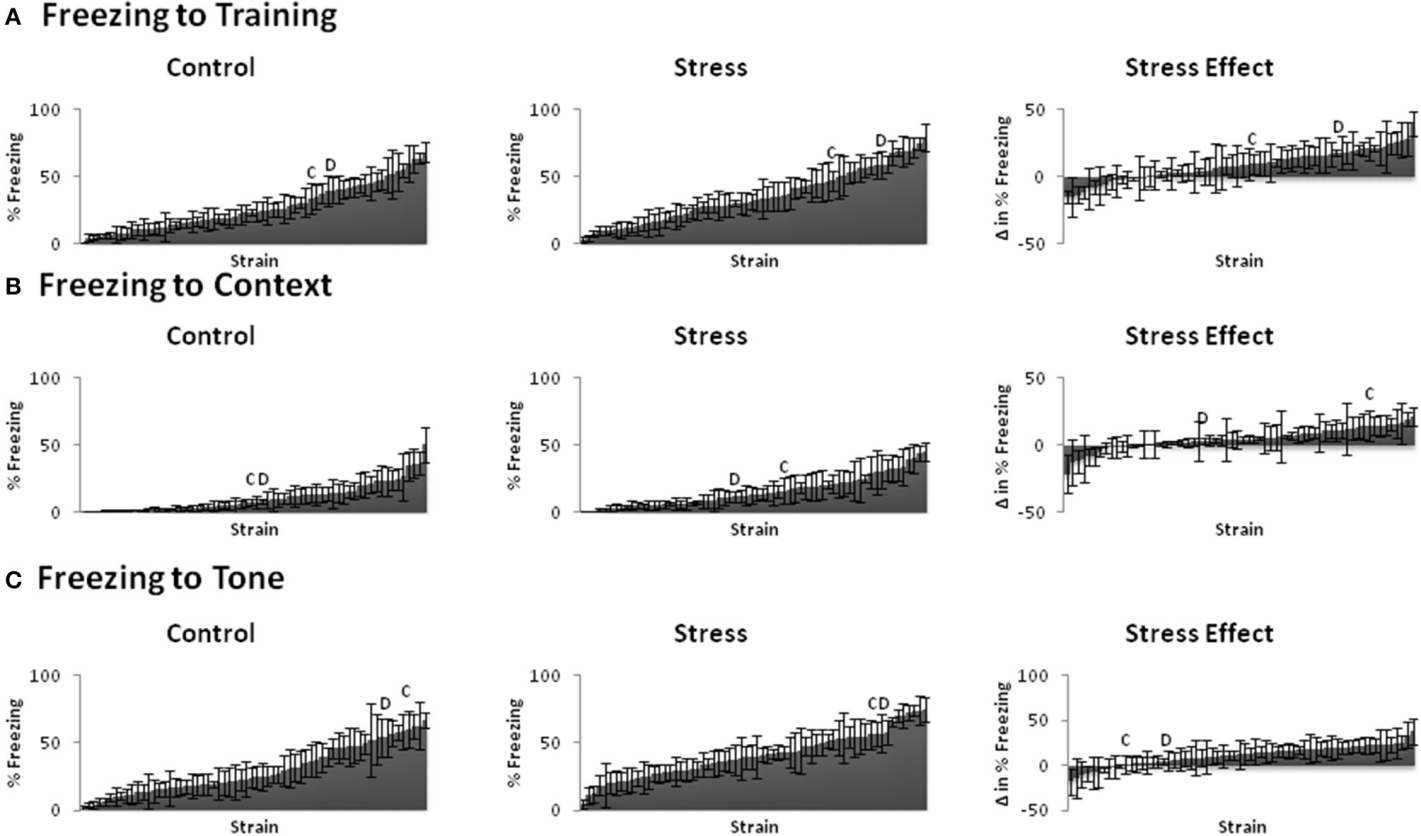
**Behavioral phenotypes of fear conditioning paradigm**. Mean ± s.e.m. % time freezing for Control (Left) and Stress (Middle) populations, and mean ± s.e.m. difference in % freezing between littermates (Stress minus Control: Stress Effect, Right) of the **(A)** Training (Day 1), **(B)** Context (Day 2), **(C)** Tone (Day 3). **C** and **D** indicate the locations of parental strains C57BL/6 and DBA/2 strains among the rankings, respectively.

**Figure 3:**
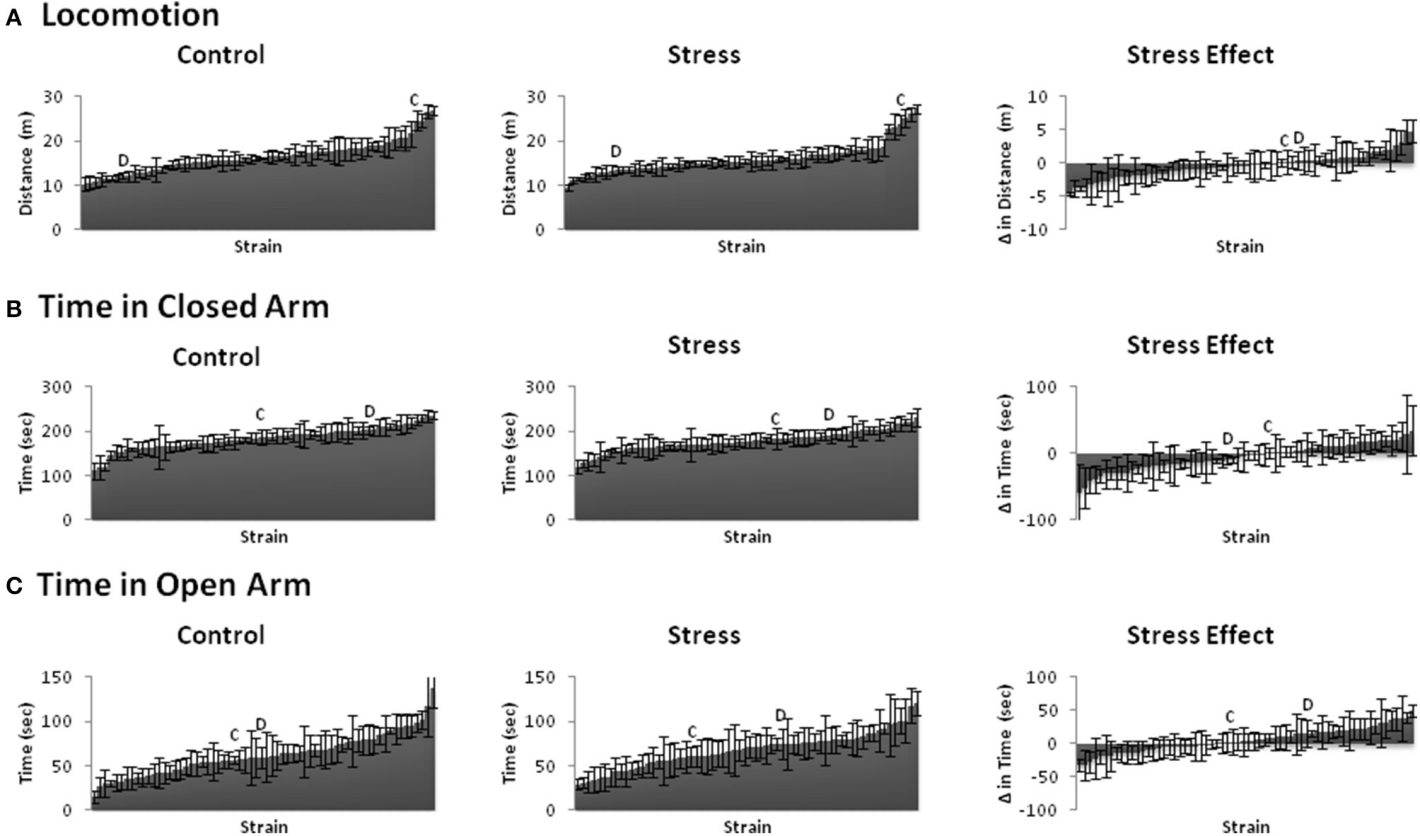
**Behavioral phenotypes of the elevated plus maze**. Mean ± s.e.m. distance or time spent for Control (Left) and Stress (Middle) populations, and mean ± s.e.m. difference in distance or time spent between littermates (Stress minus Control: Stress Effect, Right) of the **(A)** Locomotion, **(B)** Time in Closed Arm, and **(C)** Time in Open Arm. **C** and **D** indicate the locations of parental strains C57BL/6 and DBA/2 strains among the rankings, respectively.

We assessed conditioned and unconditioned emotional behavior across BXD and parental strains using the fear conditioning paradigm and elevated plus maze, respectively. Fear learning (Figure 2) and emotional behavior (Figure 3) responses varied greatly across strains. Indeed, even the trait that showed the least variation (locomotion) from lowest to highest measured response (~10–27) had a 2.5-fold difference. The susceptibility of these traits to stress-induced effects (stress-effect) was assessed by taking the difference in the measured behavior between littermates (CVS minus control). These differences ranged from negative to positive and indicate a wide variation in stress-induced effects (Figures 2, 3, right).

All emotional behavior traits were tested for correlation in both the control (Table 1) and Stress (Table 2) population. As expected, time spent in open and closed arms during the EPM was strongly negatively correlated. Traits measuring during fear conditioning shared *R* > 0.48 and *rho* > 0.54 in the control condition and *R* > 0.56 and *rho* > 0.58 in the stress population.

**Table 1:**
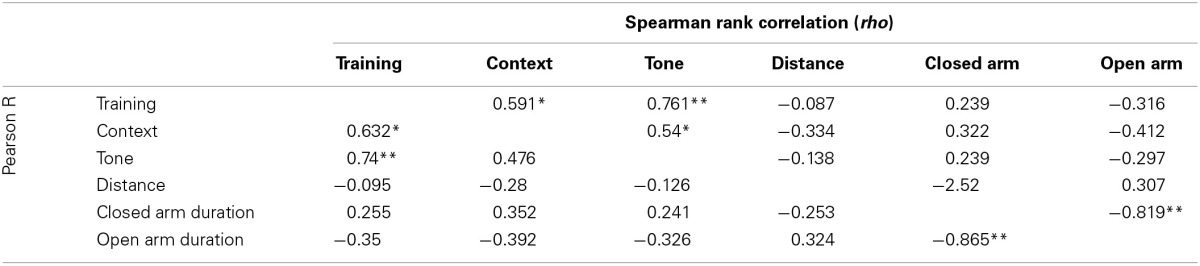
**Correlation of traits measured in control population**.

^*^Correlation values > 0.5, ^**^correlation values > 0.7.

**Table 2:**
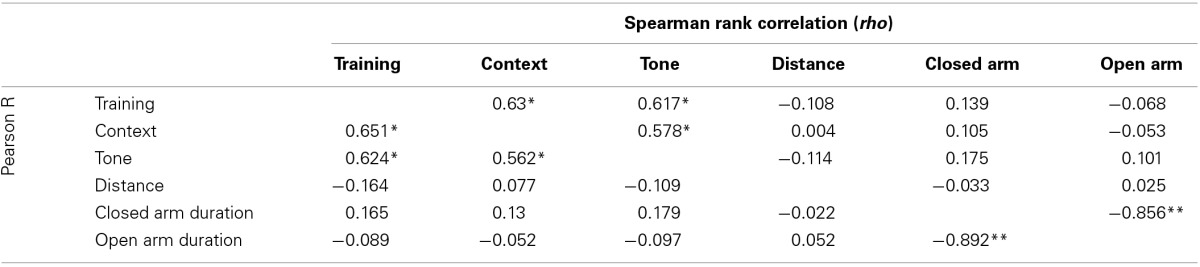
**Correlation of traits measured in stress population**.

^*^Correlation values > 0.5, ^**^correlation values > 0.7.

### Heritability of emotional behavior

We determined broad-sense and narrow-sense heritability to determine the proportion of variance across all strains for a measured trait that is attributable to genetic variance (Table 3). A *h*^2^ ≥ 0.25 indicates a strong genetic component to the trait and QTL analysis was performed on those traits with a *h*^2^ of 0.25 or greater in at least one of the populations. All measures of the fear conditioning paradigm had at least one population displaying strong heritability (*h*^2^ = 0.25–0.35). Locomotion in the elevated plus maze showed high heritability (*h*^2^ = 0.32–0.37), however time spent in closed and open arms was not determined to be strongly heritable (*h*^2^ = 0.14–0.20). Of interest, *h*^2^ of the majority of traits decreased in CVS compared to control populations.

**Table 3:** **Broad-sense (*H*^2^)/Narrow-sense (*h*^2^) heritability of behavioral traits within control and chronic stress (stress) populations and the difference in behavior between control and stress (stress minus control)**.

**Trait**	**Control *H*^**2**^/*h*^**2**^**	**Stress *H*^**2**^/*h*^**2**^**	**Stress effect *H*^**2**^/*h*^**2**^**
**FEAR CONDITIONING**			
Training	0.58/0.35	0.61/0.37	0.27/0.12
Context	0.45/0.25	0.39/0.20	0.20/0.091
Tone	0.48/0.26	0.46/0.25	0.21/0.096
**ELEVATED PLUS MAZE**			
Locomotion	0.54/0.32	0.59/0.37	0.24/0.11
Time in closed arms	0.40/0.20	0.32/0.16	0.21/0.092
Time in open arms	0.39/0.19	0.29/0.14	0.22/0.099

Broad-sense, Va/Vt; Narrow-sense, ½Va/(½Va + Vw); Va, variance among strains; Vt, variance across total population; Vw, variance within strains.

### Main effects on emotional behavior

To assess the effect of stress on emotional behaviors measured across the BXD strains, we first ran a non-linear mixed-effect test followed by ANOVA for effects of stress and strain (Table 4). An effect for strain was seen in all measures, indicating that the unique genetic background of the different strains determined the measured behavioral response, consistent with our measures of heritability. Significant effects of stress were seen in freezing to training (Day 1), context (Day 2), and tone (Day 3) in the fear conditioning paradigm and time in open and closed arms of the elevated plus maze, indicating that these behavioral responses were susceptible to chronic stress exposure. Additional environmental factors were tested as confounds in mixed model analysis (month of testing and handler during testing; Valdar et al., 2006). Month of testing did not hold a main effect for any trait tests, while handler was a significant factor in time spent in closed arms during the EPM alone *P* < 0.01 (*F* = 6.86, *df* = 3).

**Table 4:** **Summary of One-Way ANOVA results following linear mixed model fit**.

**Trait**		**Strain *P*, *F*, *df***	**Stress *P*, *F*, *df***
**FEAR CONDITIONING**			
	Training	^*^<0.001, 5.41, 63	^*^<0.0001, 16.05, 1
	Context	^*^<0.0001, 2.80, 63	^*^<0.05, 4.96, 1
	Tone	^*^<0.0001, 6.50, 63	^*^<0.0001, 26.22, 1
**ELEVATED PLUS MAZE**			
	Locomotion	^*^<0.0001, 10.15, 63	0.35, 0.92, 1
	Time in closed arms	^*^<0.0001, 2.04, 63	^*^<0.05, 4.23, 1
	Time in open arms	^*^<0.001, 1.92, 63	0.65, 0.22, 1

* indicates significant p-value.

### QTL mapping links emotional phenotypes to causal loci

**Figure 4:**
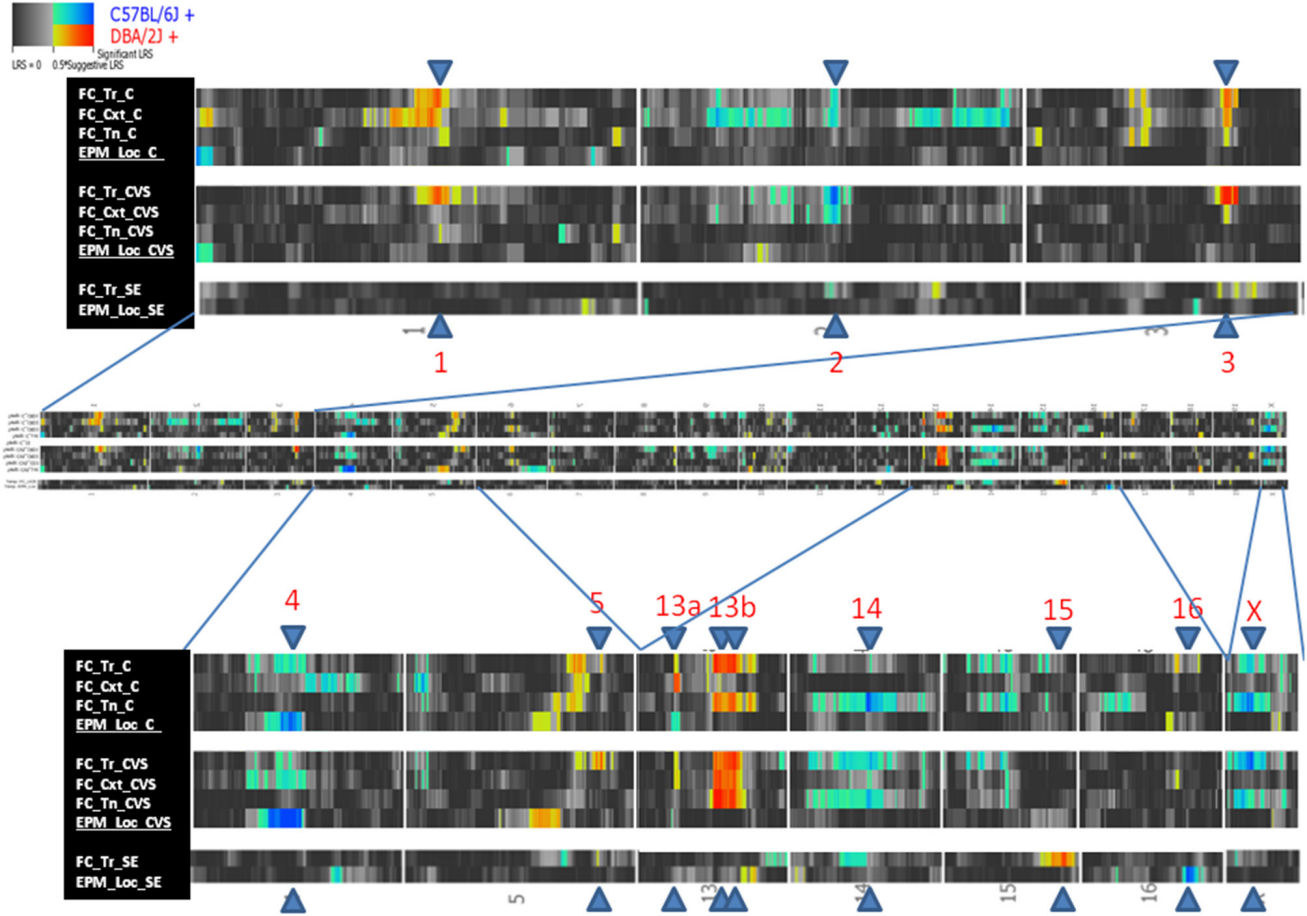
**Heatmap of QTL mapping of behavioral traits and Stress-Effect containing significant and/or suggestive peaks**. Abbreviations in legend: Fear Conditioning (FC), elevated plus maze, (EPM), freezing to training (Tr), freezing to context (Cxt), freezing to tone (Tn), locomotion (Loc), control (C), chronic variable stress (CVS), and stress-effect (SE).

**Figure 5:**
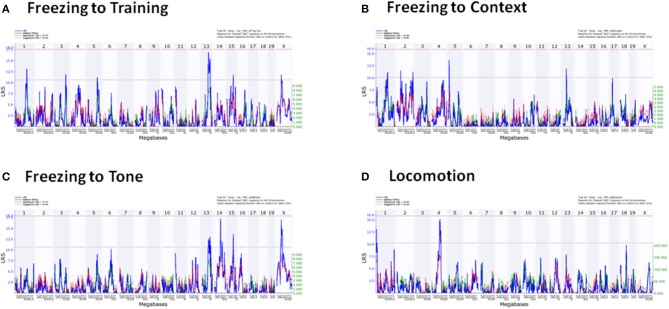
**Individual QTL maps of control population phenotypic traits**. **(A)** Freezing to Training (Day 1), **(B)** Freezing to Context (Day 2), **(C)** Freezing to Tone (Day 3), **(D)** Locomotion.

**Figure 6:**
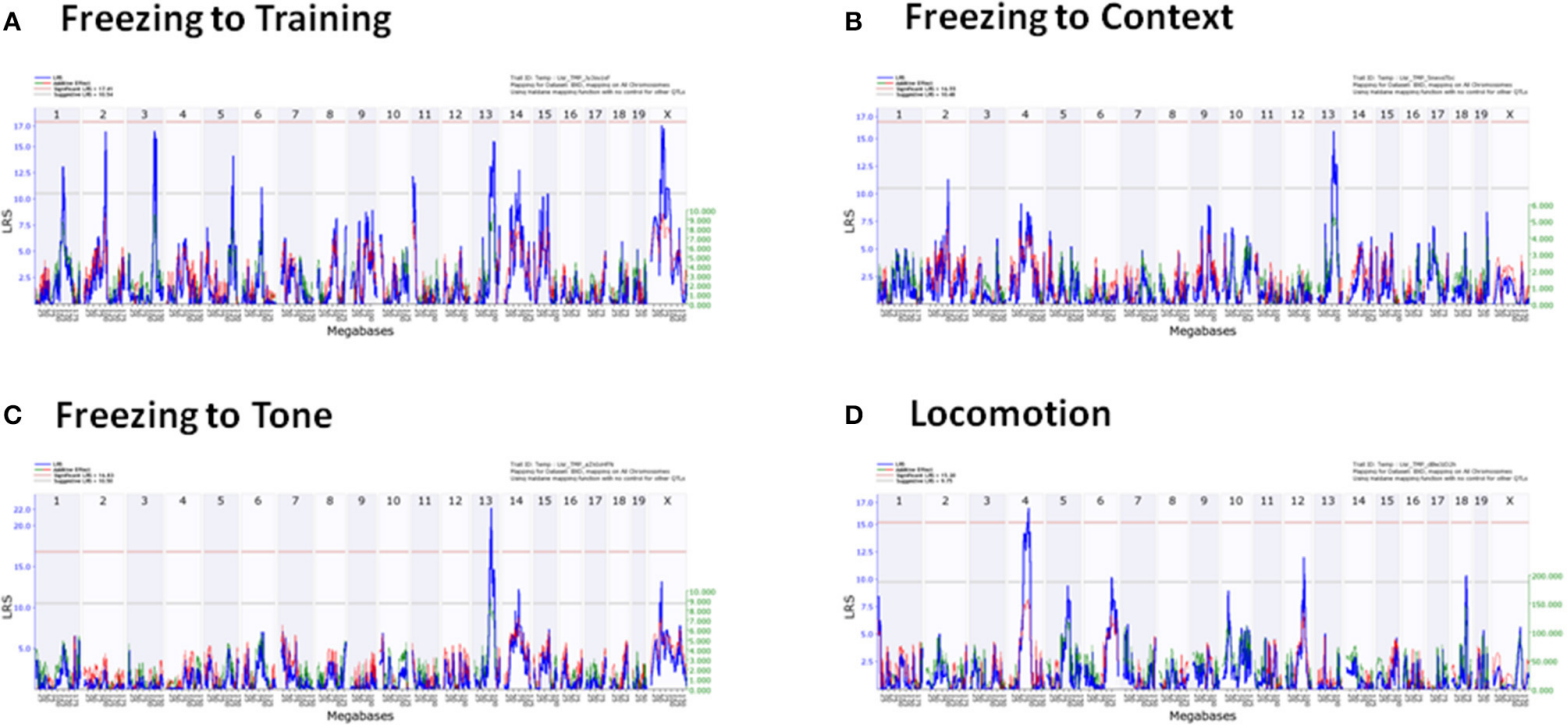
**Individual QTL maps of chronic stress population phenotypic traits**. **(A)** Freezing to Training (Day 1), **(B)** Freezing to Context (Day 2), **(C)** Freezing to Tone (Day 3), **(D)** Locomotion.

QTLs were detected in freezing to training (Day 1), context (Day 2), and tone (Day 3), and locomotion (Tables 5, 6). No QTLs meeting a suggestive level of LRS significance were found for time spent in open and closed arms of the EPM. The lack of QTLs for time spent in EPM is consistent with the non-significant *h*^2^ scores reported for these measures. Additionally, QTLs for the Stress-Effect on locomotion and freezing to training (Day 1) were detected. QTL maps of all measured traits having suggestive or significant LRS peaks are summarized in Table 3 and displayed as a heatmap (Figure 4). The use of a heatmap allows for comparison across phenotypic traits and the presence of stress. Individual QTL maps containing suggestive and significant peaks for the control and CVS populations are found in Figures 5, 6, respectively.

**Table 5:** **List of QTLs**.

**QTL**	**Trait-group**	**Chr**	**Peak LRS**	**Mapping location (Mb)**	**Genotype with increased trait**	**Previous reports for QTL (citation)**
1	Tr(C and CVS)	1	13.1^*^	117.4–126.3	DBA/2	
2	Tr(CVS)	2	16.5^*^	97.9–101.3	C57BL/6	Freezing to Context (Radcliffe et al., 2000; Parker et al., 2012); basal cort (Yang et al., 2008)
3	Tr(C and CVS)	3	16.2^*^;	118.8–124.6; 126.8–129.1	DBA/2	Freezing to Context (Owen et al., 1997)
			15.8^*^			
4	Loc(C and CVS)	4	16.5^**^	65.5–95.7	C57BL/6	Context fear conditioning (Brigman et al., 2009)
5	Tr(CVS)	5	13.3^*^	121.8–127.4	DBA/2	Contextual fear conditioning (Parker et al., 2012); Time in Open sections of zero maze (Cook, 2009 unpublished in GeneNetwork); time spent in closed quadrants following restraint (Melloni, 2009 unpublished data on GeneNetwork)
13a	Cxt(C)	13	13.7^*^	45.8–50.0	DBA/2	Contextual learning (Philip et al., 2010); glial count in amygdala (Mozhui et al., 2007)
13b	Tr(C and CVS), Tn(C and CVS), Cxt(CVS)	13	22.2^***^	78.1–97.5	*DBA/2*	Hippocampal volume (Philip et al., 2010); freezing during fear conditioning (Lassalle et al., 1994); hippocampal activation (Enoch et al., 2008)
14	Tn(C)	14	17.3^**^	71.0–73.5	C57BL/6	
15	Tr(SE)	15	13.7^*^	93.2–95.4	DBA/2	
16	Loc(SE)	16	17.0^**^	74.9–79.1	C57BL/6	
X	Tr(C and CVS), Tn3(C and CVS)	X	17.0^**^	56.2–68.3	C57BL/6	Hippocampal mossy fiber CA4/total MF (Lassalle et al., 1994)

Trait/group abbreviations: C, control; CVS, chronic variable stress; Chr, chromosome; SE, stress-effect; Tr, freezing to training; Cxt, freezing to context; Tn, freezing to tone; and Loc, locomotion.

LRS: greatest LRS reported. Significant level:

* suggestive,

** significant,

*** highly significant per http://genenetwork.org.

**Table 6:** **List of candidate genes**.

**QTL**	**Chr**	**# Genes (candidate/total)**	**Candidate genes**
1	1	20/32	Cntnap5a^^∧^^, Tsn^^∧^^, Mki67ip, Clasp1, Gli2, Inhbb, Ralb^^∧^^, Ptpn4, Sctr, Tmem37^^∧^^, Dbi^^∧^^, 3110009E8Rik^^∧^^, C1ql2^^∧^^, Marco, En1, B230209K01Rik^^∧^^, Insig2, Htr5b^^∧^^, Ddx18, Dpp10
2	2	0/1	
3	3	30/55	Dpyd, Ptbp2^^∧^^, Rwdd3^^∧^^, Tmem56^^∧^^, Alg14^^∧^^, Cnn3^^∧^^, Scl44a3^^∧^^, F3^^∧^^, Abcd3^^∧^^, Arhgap29^^∧^^, Abca4^^∧^^, Gclm^^∧^^, Dnttip2^^∧^^, Bcar3^^∧^^, Fnbp1l^^∧^^, Pde5a^^∧^^, Fabp2, Usp53^^∧^^, Myoz2^^∧^^, Sec24d^^∧^^, 2310068J10Rik^^∧^^, G430022H21Rik^^∧^^, Synpo2^^∧^^, Prss12^^∧^^, Ndst3; Neurog2, Alpk1, Alpk1, Pitx2, Enpep^^∧^^
4	4	60/115	Astn2, Tlr4, Dbc1, Cdk5rap2, C630043F03Rik^^∧^^, Tle1, Rasef, Frmd3^^∧^^, Jmjd2c^^∧^^, 3110001D03Rik^^∧^^, Ptprd^^∧^^, Tyrp1^^∧^^, Mpdz^^∧^^, Nfib^^∧^^, Zdhhc21^^∧^^, Cer1, Frem1, 1810054D07Rik^^∧^^, Snapc3^^∧^^, Psip1^^∧^^, 4930473A06Rik^^∧^^, 8430420F16Rik^^∧^^, Cntln^^∧^^, Sh3gl2^^∧^^, AdamtsI1^^∧^^, Rraga, 6230416J20Rik^^∧^^, Adfp, Rps6^^∧^^, Asah3l^^∧^^, Slc24a2^^∧^^, Mllt3, Ifnb1, Ifna14, Ifna13, Ifna2, Klhl9^^∧^^, Ifna7, Ifna11^^∧^^, Ifna6, Ifna5, Ifna4, Ifna1, Ifne1, Mtap^^∧^^, Cdkn2a, Cdkn2b^^∧^^, Dmrta1, Elavl2, Tusc1, 5830433M19Rik^^∧^^, Plaa^^∧^^, Ift74^^∧^^, Lrrc19, Tek^^∧^^, 4930579C15Rik^^∧^^, Mysm1^^∧^^, Jun, 9530080O11Rik^^∧^^, Hook1
5	5	57/112	Trafd1, Mapkap5, Aldh2, Acad10, Brap^^∧^^, Atxn2, Myl2, Ppp1cc, Rad9b, Vps29^^∧^^, Arpc3, Anapc7, Atp2a2, P2rx7, P2rx4, Camkk2, Anapc5, Rnf34, Fbxl10, Hpd, Psmd9^^∧^^, Wdr66^^∧^^, Bcl7a, Mixip^^∧^^, Il31, Lrrc43, B3gnt4, Diablo^^∧^^, Vps33a, Clip1^^∧^^, Zcchc8, Kntc1^^∧^^, Gpr109a, Gpr81, Denr, Hip1r, Abcb9, Arl6ip4, Pitpnm2, Mphosph9^^∧^^, Cdk2ap1^^∧^^, Sbno1^^∧^^, Ddx55^^∧^^, Eif2b1, Gtf2h3, Atp6v0a2, Ccdc92^^∧^^, Zfp664^^∧^^, 3110032G18Rik^^∧^^, Ncor2, Ubg^^∧^^, Scarb1^^∧^^, Ubc^^∧^^, Dhx37^^∧^^, Bri3bp^^∧^^, Aacs, Tmem132b^^∧^^,
13a	13	27/57	Atxn1^^∧^^, 5033430I15Rik^^∧^^, Rbm24, Cap2, C78339^^∧^^, Up153, Kif13a^^∧^^, Nhlrc1^^∧^^, Tpmt^^∧^^, Aof1^^∧^^, Dek^^∧^^, Id4, A330048O09Rik^^∧^^, Ptpdc1, Phf2, Wnk2^^∧^^, Ninj1, Susd3, Fdg3, Bicd2, Cenpp^^∧^^, Ecm2, Aspn. Omd, Ogn, Nol8, Iars
13b	13	52/100	A430105P17Rik^^∧^^, C130051F05Rik^^∧^^, Arrdc3^^∧^^, Cetn3, Mef2c^^∧^^, Tmem161b^^∧^^,Ccnh^^∧^^, Rasa1^^∧^^, Cox7c^^∧^^, Edil3^^∧^^, Hapln1^^∧^^, Van^^∧^^, Xrcc4, Rps23, Atg10^^∧^^, Ssbp2^^∧^^, Acot12^^∧^^, Zcchc9, Ckmt2, Rasgrf2, Msh3, Dhfr, Zfyve16, Spz1, Thbs4^^∧^^, EG218444^^∧^^, Papd4, Mtx3, Homer1^^∧^^, Jmy^^∧^^, Cmya5^^∧^^, Bhmt, Bhmt2, Dmgdh, Arsb^^∧^^, Lhfpl2, Scamp1^^∧^^, Ap3b1, Tbca, Otp, Wdr41, Pde8b, Zbed3, Crhbp, S100z, F2rl1^^∧^^, F2r^^∧^^, Iqgap2^^∧^^, F2rl2^^∧^^, Sv2c^^∧^^, Pol^^∧^^, Col4a3bp^^∧^^
14	14	6/15	Fgf17, Npm2^^∧^^, Xpo7^^∧^^, Dok2^^∧^^, Gfra2^^∧^^, Cysltr2
15	15	9/12	Zcrb1^^∧^^, Pphln1^^∧^^, Prickle1^^∧^^, D630014N10Rik^^∧^^, Pus7l^^∧^^, Irak4^^∧^^, Twf1^^∧^^, Tmem117^^∧^^, Nell2^^∧^^
16	16	14/29	E130102B10Rik^^∧^^, Lipi, Rbm11^^∧^^, Stch^^∧^^, Samsn1^^∧^^, Nrip1^^∧^^, Usp25^^∧^^, 2810055G20Rik^^∧^^, D130020G16Rik^^∧^^, Cxadr^^∧^^, Btg3^^∧^^, 4930578L05Rik^^∧^^, Chodl, Prss7
X	X	14/39	Fgf13, F9, Mcf2, Atp11c^^∧^^, Sox3, Ldoc1, Ctag2^^∧^^, 383047A13Rik^^∧^^, Slitrk4, Fmr1, Fmr1nb, 2610007B07Rik^^∧^^, Ids, Fate1^^∧^^

Candidate genes were defined as genes with a human homolog and/or are cis-regulated:

∧ indicates cis-regulated gene.

For reference, QTLs are denoted by chromosome location (Table 5). QTLs unique to the control population were located on Chromosomes 13 and 14 (QTLs 13a and 14) and were found in the analysis of freezing to context (Day 2) and tone (Day 3), respectively. Conversely, QTL mapping of freezing to training (Day 1) uncovered QTLs unique to the CVS population on Chromosomes 2 and 5. All other QTLs (QTLs 1, 3, 4, 13b, and X) were shared by both control and CVS populations. QTLs 1, 3, and 13b were identified in both control and CVS populations for freezing to training. QTL 13b was also identified in freezing to context (Day 2) for both control and CVS populations and freezing to tone (Day 3) in the CVS population. Lastly, QTL 4 was identified in both control and CVS populations for locomotion in the elevated plus maze. Candidate genes within each QTL are listed in Table 6.

## Discussion

We observed significant effects of chronic stress on emotional behavioral phenotypes. The BXD populations utilized revealed great variability of behavioral response in both control and CVS conditions (Figures 1, 2). Thus, our results show that environmental stress is a unique factor affecting behavioral responses in BXD strains of mice.

QTL mapping of individual phenotypes identified genetic loci that predict emotional behavior in control and stress environments (Table 5). The unique QTL maps from control vs. chronic stress populations for the same behavioral attribute suggest that the genetic regions most likely to predict behavior are influenced by prior stress history. Of interest, the strength of each trait's heritability score lessened with the presence of chronic stress, suggesting a disruption of the basal heritable contribution with chronic stress. Our mixed-model analysis identified behavioral traits susceptible to stress-induced effects that were seen in differences in their resulting QTL maps (Table 4). Most QTLs for the behavioral phenotypes reported here have not been previously indentified for these traits, although several have been identified for related phenotypes (Table 5). As the initial report of these QTLs and candidate genes, additional studies are required to strengthen their validation.

### QTLs and candidate genes for learning within a stressful context

QTL 13b was found repeatedly for fear learning in both control and chronic stress populations. Although its strength and frequency is greater with chronic stress, the region was not identified for anxiety/exploratory EPM behavior. Its presence in freezing to training (Day 1) and tone (Day 3) of the control population suggests pleiotropic effects for learning within a stressful context. QTL 13b corresponds with peaks found previously for fear conditioning and hippocampal volume (Table 5; Lassalle et al., 1994; Philip et al., 2010; Parker et al., 2012). The myocyte-specific enhancer binding factor 2C (*Mef2c*) gene resides at the highest LRS value within QTL 13b, and is cis-regulated. Gene expression of *Mef2c* in the amygdala has been associated with anxiety (Genenetowrk GeneWiki: Williams et al., 2001), and plays a role in synapse elimination within the hippocampus to facilitate hippocampal dependent learning (Barbosa et al., 2008). Additionally, Mef2c has been identified as a requirement for the activation-dependent expression of BDNF (Lyons et al., 2012) and is a marker of ischemia-resistant hippocampal neurons (Speliotes et al., 1996). Mutations of *Mef2c* in humans result in mental retardation (Zweier et al., 2010). Taken together, its presence in QTL 13b and its known associations suggest that it may have a role during stress and/or anxiety.

Relevant to learning and stress, corticotropin-releasing hormone binding protein (*Crhbp*) is also found within QTL 13b. *Crhbp* regulates the activity of CRH (corticotropin releasing hormone), a stress hormone in the HPA axis (Westphal and Seasholtz, 2006), and is upregulated following stress (McClennen et al., 1998). The HPA axis has a complex relationship with learning and memory- transient activation results in enhancement of learning and memory (de Kloet et al., 1999), while persistent activation levels results in cognitive deficit (de Kloet et al., 2005). Enoch et al. reported that dense whole genome linkage scan of hippocampal activation assessed by EEG resulted in a linkage peak containing *crhbp* (Enoch et al., 2008). *Crhbp* is found at greater levels in high avoidance rats compared to low avoidance rats (Sabariego et al., 2011). Additionally, mutations in *Crhbp* were associated with anxiety disorders in a Plains Indian population and alcohol use in Caucasians, suggesting a role for *Crhbp* in stress-induced phenotypes (Enoch et al., 2008). These results suggest *Crhbp* may be a marker for fear learning in a stress context.

Also associated with fear learning in both control and chronic stress populations, QTL X was identified for the freezing to training and tone (Days 1 and 3) and absent for context (Day 2). Genes within this region may play a role in the incorporation of the auditory cue and aversive stimulus. Fibroblast growth factor 13 (*Fgf13*) is located at the LRS peak of QTL X and has a well described role in learning and memory (Wu et al., 2012). Expressed in cortical neurons*, Fgf13* is linked to X-chromosome-linked mental retardation in humans (Itoh and Ornitz, 2008). Interestingly, the expression of the *Mef2c* (QTL 13b) and *Fgf13* in the hippocampus are positively correlated (genenetwork.org, *Rho* = 0.543, *P* = 0.00356). Thus, genes in both QTLs 13b and X may interact for the expression of learning phenotypes.

QTL 5 was unique to the stress population for freezing to training (Day 1) and has been found previously (Table 5; Parker et al., 2012; Cook, 2009 unpublished data in genenetwork.org) Within QTL 5, *P2rx7*, and *P2rx4* have roles in neuroinflammatory response to stress (Witting et al., 2004; Hernandez et al., 2010). Knockout and antagonism of *P2rx7* results in mood stabilizing and reduces the corticosterone response to restraint stress (Csolle et al., 2013). Calcium/calmoudulin kinase kinase 2 (*Camkk2*), a gene associated with hippocampus-dependent long-term memory and anxiety also resides in QTL 5 (Peters et al., 2003; Mizuno et al., 2007; Sabariego et al., 2011). Loss of *Camkk2* decreases BDNF expression (Kokubo et al., 2009). Interestingly, fear conditioning results in a downregulation of *Camkk2* in the hippocampus (Mei et al., 2005) and *Camkk2* knock outs males, but not females, show impaired spatial learning and normal fear conditioning (Peters et al., 2003; Mizuno et al., 2007). Our experiments, using only male BXD mice, confirm a contributory role of Camkk2 in fear learning.

QTL analysis of the Stress-Effect revealed a significant peak for difference in locomotion (QTL 16) and suggestive peak for difference in freezing to training (Day 1) (QTL 15). These genetic regions contain Nrip1 and Nell2, two genes required for learning of the Morris Water Maze (Duclot et al., 2012) (Matsuyama et al., 2005). Of note, swimming aberrations were noted as a potential contributor to the cognitive deficit, despite the absence of difference in rotorod and open field performance (Duclot et al., 2012). Elimination of *Nell2* via knockout results in increases in LTP within the dentate gyrus (Matsuyama et al., 2004), suggesting that its role in cognitive performance is due to a regulatory effect on LTP.

### QTLs and candidate genes for learning in a control environment

Unique to learning in the control environment, QTLs 13a and 14 expectedly contained genes associated with learning. No genes with established rolesin stress or anxiety were found. QTL 13a, previously found for contextual learning and amgdala glial count (Table 5; Mozhui et al., 2007; Philip et al., 2010) contains NHL repeat containing 1/epilepsy, progressive myoclonic 2B (*Nhlrc1/EPM2B*). Knockout of this gene results in episodic memory deficits assessed by the object recognition test (Garcia-Cabrero et al., 2012). Thus, *Nhlrc1* may play a role in the incorporation or retrieval of contextual memory. Learning an auditory cue (freezing to tone- Day 3) resulted in a significant QTL on Chromosome 14 in controls. Several genes within this locus are associated with hippocampal-dependent learning [e.g., *Gfra, Itm2B*, and *Htr2a* (Voikar et al., 2004; Tamayev et al., 2010; Zhang et al., 2013)]. Loss of the QTLs with chronic stress may indicate that genes within QTLs 13a and 14 no longer play a significant role when stress is present, or that genes within other genetic loci play overshadowing roles with stress. Of note, the expression of *Nhlrc1* in QTL 13a, and *Itm2b* and *Htr2a* in QTL 14 in the whole brain correlate positively with whole brain expression of the gene *Slc24a2* in QTL 4 (genenetwork.org, *Rho* = 0.612, *p* < 0.00037, *Rho* = 0.549, *p* < 0.0020, and *Rho* = 0.581, *p* < 0.00089, respectively). This is of interest because these four genes have been associated with learning and may act in concert to affect learning in their respective phenotypes.

The results from this study identified genetic loci for emotional behavioral phenotypes in the presence and absence of chronic stress. Four of the loci identified in this study are novel and their presence across multiple traits supports further study of the candidate genes contained within. In sum, our data indicate that distinct genetic loci are associated with emotional behavior in control and chronic stress conditions suggesting that behavioral outcomes are influenced by the interplay between prior stress history and genetic background.

### Conflict of interest statement

The authors declare that the research was conducted in the absence of any commercial or financial relationships that could be construed as a potential conflict of interest.
